# The association between childhood adversity and the conserved transcriptional response to adversity (CTRA) in sexual minority men

**DOI:** 10.21203/rs.3.rs-2585046/v1

**Published:** 2023-02-14

**Authors:** Shareefa Dalvie, Michael Li, Mariah Kalmin, Steven Cole, Dan Joseph Stein, Steven Shoptaw

**Affiliations:** South African Medical Research Council; UCLA: University of California Los Angeles; UCLA: University of California Los Angeles; UCLA: University of California Los Angeles; UCT: University of Cape Town; UCLA: University of California Los Angeles

## Abstract

Adverse childhood experiences (ACES) increase risk for mental and physical health disorders in adulthood, particularly in individuals from sexual and ethnic minority groups. The effects of ACES on health may be mediated by the immune system. The exact mechanisms by which an environmental exposure, such as childhood adversity, can affect the immune system are still unknown. The aim of this study was to determine whether early adversity predicts significant changes in the expression of a predefined set of immune–related genes, known as the conserved transcriptional response to adversity (CTRA), in a diverse group of sexual minority men (SMM). Participants included HIV positive and negative males from the mSTUDY. Expression data from 53 CTRA genes were obtained at baseline and 12-month follow-up. Childhood adversity was measured with the 10-item ACES questionnaire. Wilcoxon rank sum and chi-squared tests were used to assess differences in sociodemographic variables and HIV status between exposed (cumulative ACES ≥ 3) and unexposed groups (cumulative ACES ≤ 2). Linear mixed models were used to determine associations between ACES (cumulative score, dichotomous measure and subscales) and CTRA gene expression. There were no differences in age and employment status between the exposed and unexposed groups. A larger number of exposed participants were HIV positive than in the unexposed group (p = 0.03). There were no significant associations between any of the ACES variables and CTRA gene expression. A range of factors may have contributed to this unexpected finding. Further studies are needed to assess the biological effects of ACES in adulthood.

## Introduction

Adverse childhood experiences (ACES) are highly pervasive with a prevalence of approximately 39% across high-, middle- and low-income countries^1^. Due to structural inequities, individuals from minority groups experience even higher ACE exposure compared to others in the population. For example, the prevalence of ACES in gay, bisexual, and other sexual minority men (SMM) is approximately 80%^2^. Further, this prevalence is even greater in individuals who belong to multiple minority groups (e.g., both a sexual *and* ethnic minority group)^3^.

ACES encompass all forms of abuse (physical, sexual and emotional), neglect (physical and emotional), and household dysfunction (e.g., living with an alcoholic or substance abuser or having a household member imprisoned) experienced before 18 years of age^4^. These early traumatic life events can have far-reaching effects as they increase the risk for both mental and physical health disorders in adulthood^5,6^. For example, ACES have shown to be associated with poorer mental health outcomes in individuals with HIV^7^.

Individuals from sexual and ethnic minority groups have shown to have a higher prevalence of physical and mental health disorders compared to the general population^8–10^. This higher prevalence may be explained by the minority stress model which posits that individuals from minority groups experience a stressful social environment due to several factors such as low socioeconomic status, discrimination and prejudice, which in turn results in poorer mental health^11^. Besides the stressors experienced during adulthood, adverse social experiences during childhood have also shown to increase the risk of psychological distress, substance use, and HIV infection during adulthood in SMM^2,12,13^.

The effects of ACES on health may be mediated by the immune system^14^. For example, both prospective and retrospective reports of ACES were associated with increased levels of inflammatory markers during mid-life in a birth cohort^15^. Another study showed that participants who had experienced physical, sexual or emotional abuse before 18 years of age, had a steeper increase in inflammation levels over time, compared to participants without a history of childhood abuse^16^. These findings suggest that ACES have long-lasting effects on the immune system. Moreover, childhood adversity was shown to be associated with differential expression of genes involved in the immune system in adults^17^.

Previous research has shown that exposure to threat is associated with a distinct immune-related gene expression pro le, known as the common conserved transcriptional response to adversity (CTRA). The CTRA pro le is characterized by up-regulation of the expression of genes involved in inflammation and down-regulation of genes involved in type I interferon responses and antibody synthesis^18^. This pattern of gene expression was demonstrated in 4-month-old rhesus macaques who had experienced adverse social environments (being surrogate/peer-reared vs maternally reared)^19^. In humans, ACES were associated with increased expression of both inflammatory and interferon genes in women with breast cancer who had experienced childhood maltreatment^20^. This gene expression pattern was also observed in a sample of 37 children with exposure to ACES^21^ and former child soldiers^22^.

The exact mechanism by which an environmental exposure, such as childhood adversity, is able to affect our immune system is mostly unknown. Therefore, the aim of this study was to determine whether early adversity predicts significant changes in the expression of a predefined set of immune–related genes during adulthood in an ethnically diverse group of SMM. By investigating the relationship between childhood adversity and CTRA expression pro le, we hope to provide insight into the biological effects of adverse environmental exposures.

## Methods

### Study Participants

This study used data collected from the mSTUDY (*Men Who Have Sex with Men and Substance Use Cohort at UCLA Linking Infections, Noting Effects*), an ongoing cohort study for which a broad aim is to understand the effects of substance use on the immune system of HIV positive and negative SMM. This study based in Los Angeles (USA), first started recruiting in 2014, and comprises participants from diverse backgrounds. Participants undergo laboratory testing, bio-specimen collection, physical examination, and computer-based survey assessments (which includes questions on psychosocial, behavioral, and physical health) every 6 months^23^. In the current study, we used data from 131 African American and 128 Latino SMM. This study received ethical approval from the SAMRC Human Research Ethics Committee (EC050–11/2021).

### Transcriptional Pro ling

Details regarding the transcriptional pro ling are outlined in^24^. Brie y, peripheral blood mononuclear blood cells (PBMCs) were obtained from 125ml blood samples. RNA was isolated from PBMCs using Qiagen RNeasy, quantified using RiboGreen and the integrity verified using the Agilent TapeStation. RNA samples were converted to cDNA (Lexogen QuantSeq 3’ FWD) and sequenced on an Illumina HiSeq 4000 instrument at the UCLA Neuroscience Genomics Core Laboratory, using standard protocols. The average read per sample was 15.1 million. These reads were mapped to the consensus human transcriptome and normalized to transcripts per million and log_2_ transformed^25^. Transcriptional data were from two timepoints: baseline and 12-month follow-up.

### Adverse Childhood Experiences

Childhood adversity was measured with the 10-item Adverse Childhood Experiences (ACE) questionnaire^26^ administered using a computer-assisted self-interview. Participants were asked about adverse experiences before the age of 18 years (physical abuse, emotional abuse, sexual abuse, emotional neglect, physical neglect, and household dysfunction) with “no or “yes” responses to each question. “Yes” and “no” answers were coded as 1 and 0, respectively. An ACES cumulative score was generated, a sum of the 10 ACE items. From this, a binary ACES variable was created where participants with more than 2 ACES were considered as “exposed” and anyone with less than or equal to 2 ACES were coded as “unexposed”. We also assessed childhood maltreatment (abuse and neglect subscales together) and each abuse and neglect subscale separately (binary variables), and household dysfunction subscales.

### Statistical Analysis

The Wilcoxon rank sum test for independent samples was used to determine whether there were differences in age between the exposed and unexposed groups. Chi-squared tests were used to determine differences in HIV status, employment, smoking, weekly alcohol use and methamphetamine use between exposed and unexposed groups.

A power calculation was conducted using the R package “*powerEQTL*”. A sample size of 9 participants, with data at two time-points, would be needed to achieve 80% statistical power, with a standard deviation of 1 for the outcome (53 CTRA genes), a family-wise Type I error rate of 0.05, and an intra-class correlation of 0.8.

Only genes with expression values of greater than or equal to 1 standard deviation were retained for analysis. Therefore, of the total 53 CTRA genes, we included 47 genes (28 interferon (IFN) genes and 19 inflammatory genes). A list of the CTRA genes included in the analysis can be found in **Supplementary File 1**. Both the inflammatory genes and the IFN genes were included in the same analysis, where the IFN gene data were negatively weighted to reflect the inverse role of IFNs in the CTRA pro le overall. We also assessed each gene set (inflammatory and IFN) in separate models. Initially, multivariable linear mixed models were used to test associations between CTRA gene expression and potential covariates, which included age, ethnicity, HIV status, weekly alcohol consumption, smoking, and methamphetamine use. Visit number and gene were included as fixed effects and participant ID was included as a random effect to account for within-person correlations in the model. The default unstructured variance-covariance matrix was used. Any variables obtaining a p-value < 0.05 were included as covariates in the final linear mixed model assessing the association between ACES and CTRA gene expression. All analyses were conducted in the statistical environment R (http://www.r-project.org/).

## Results

### Participant Characteristics

Sociodemographic characteristics of the participants are listed in Table 1. The ACES scores ranged from 0–10 and the median score was 2. For the binary ACES score, 131 participants were considered unexposed and 128 were considered as being exposed to childhood adversity. When considering the abuse and neglect subscales, emotional abuse was reported the most (41.7%) and physical neglect the least (13.5%). There were no differences in age and employment status between the exposed and unexposed groups (Table 2). However, there were significant differences in HIV status between the exposed and unexposed groups (p = 0.03). A larger number of the exposed participants were HIV positive than the unexposed group (Table 2).

### Associations between ACES and CTRA gene expression

Age, HIV status, and methamphetamine use were significantly associated with CTRA gene expression (p-value < 0.05) (**Supplementary Table 1**) and were included as covariates in the final model testing for association between ACES and CTRA. There were no significant associations between childhood adversity (including the binary variable, childhood maltreatment and each of the subscales) and CTRA gene expression for both the combined and separated models (Table 3, Supplementary Tables 2–3).

## Discussion

In this study, we sought to assess whether adversity during early life was associated with expression changes in a set of immune-related genes in an ethnically diverse group of SMM. We did not find any significant relationships between a total ACES score, a binary ACES variable, childhood maltreatment or ACES subscales, with CTRA gene expression. Further, we observed that participants with childhood adversity were more likely to be HIV positive than those without early life adversity.

We observed that participants who reported childhood adversity were more likely to be living with HIV than those who were unexposed. This may suggest that childhood adversity increases behaviors that convey a risk for HIV infection. This is in line with previous research showing that individuals exposed to abuse and neglect had a higher incidence of HIV^27^ and increased HIV-risk behaviors^28^. Thus, the prevention of childhood adversity should be considered a priority when considering HIV prevention^27^. We have also showed here and in previous work by our group that HIV status and HIV viral load is associated with CTRA gene expression in African American and Latino SMM^25^. Therefore, it appears that both ACES and CTRA gene expression have associations with HIV status but whether a relationship between early adversity and the immune system exists in our target population group, remains unclear.

Our findings suggest that adversity experienced during childhood in African American and Latino SMM may not be associated with the specific gene expression pro le characteristic of CTRA. This is similar to previous studies which found that early adversity did not influence the expression of 32 inflammatory-related genes in healthy controls^29^ and was not associated with immune markers of inflammation in a Native American population group^30^. However, sexual minority stress (comprising measures of concealment, internalised stigma and rejection from others) experienced during adulthood was associated with gene expression changes in inflammatory and immune-related function in HIV positive SMM^31^. Further, previous work by our group showed that in HIV negative SMM, homophobic victimization was associated with upregulated CTRA gene expression^24^. We may speculate that in ethnic and sexual minority groups only adverse events and stressors experienced during adulthood is associated with immune system dysregulation.

This study has several limitations. First, childhood adversity was measured using a retrospective, self-report of early life events. Thus, the exposure of childhood adversity in this study may have been influenced by recall bias and the participants’ subjective opinion of early life events^32,33^. Further, prospective and retrospective reports of childhood adversity have shown to have low agreement^34^. Second, gene expression levels were measured in peripheral tissue. Given the psychological impact of adversity, brain tissue may be more relevant to investigate for this exposure and future studies should utilize postmortem brain tissue for transcriptional pro ling. Third, we only assessed gene expression in an *a priori* set of immune related genes which may not be relevant in these study populations. With larger sample sizes we will be powered to assess the effects of early adversity on the entire transcriptome, in an hypothesis free manner.

Findings from this study suggest that early adversity may not have an association with the expression of CTRA genes in our study population but could potentially increase HIV behavioral risk factors. Further studies are needed to assess the biological effects of ACES during adulthood in minority groups.

## Figures and Tables

**Table 1:** Sociodemographic Characteristics of mSTUDY Participants (Total n = 259)

Variable	Median	Range
**Age**	31 years	19–46 years
**Total ACES score**	2	0–10
**Childhood maltreatment**	1	0–5
**Household dysfunction**	1	0–5
	Number	Percentage (%)
**Ethnicity- African American**	131	50.6
**ACES exposed**	128	49.4
**Physical Abuse exposed**	91	35.1
**Sexual Abuse exposed**	74	28.6
**Emotional Abuse exposed**	108	41.7
**Emotional Neglect exposed**	80	30.1
**Physical Neglect exposed**	35	13.5
**HIV Positive**	135	52.1
**Employed (full time and part-time)**	135	52.7

**Table 2:** Differences in sociodemographic characteristics, smoking and substance use between ACES Exposed and Unexposed groups

Variable	Median/N		P-value
	Unexposed (n = 131)	Exposed (n = 128)	
Age	30 years	32 years	0.23
**HIV status**			
Positive	59	76	**0.03**
Negative	72	52	
**Employment Status**			
Employed	60	61	0.91
Unemployed	69	66	
**Smoking**			
No	70	72	1
Yes	49	52	
**Weekly alcohol use**			
No	95	87	0.86
Yes	36	36	
**Methamphetamine Use**			
No	106	99	0.66
Yes	25	28	

**Table 3:** Results of linear mixed models of ACES total score, ACES binary score, childhood maltreatment and ACES subscales with CTRA gene expression (combined gene sets)

Predictor	Estimate	Standard error	p-value
**ACES - total score**	0.002	0.013	0.888
**ACES - binary measure**	0.043	0.077	0.572
**Childhood maltreatment**	0.009	0.021	0.659
**Household dysfunction**	−0.006	0.025	0.800
**Physical Abuse**	−0.042	0.078	0.595
**Sexual Abuse**	0.052	0.083	0.527
**Emotional Abuse**	−0.010	0.077	0.895
**Emotional Neglect**	0.101	0.080	0.207
**Physical Neglect**	−0.161	0.092	0.083
